# Nuclear microRNA-466c regulates *Vegfa* expression in response to hypoxia

**DOI:** 10.1371/journal.pone.0265948

**Published:** 2022-03-31

**Authors:** Pia Laitinen, Mari-Anna Väänänen, Ida-Liisa Kolari, Petri I. Mäkinen, Minna U. Kaikkonen, Marc S. Weinberg, Kevin V. Morris, Paula Korhonen, Tarja Malm, Seppo Ylä-Herttuala, Thomas C. Roberts, Mikko P. Turunen, Tiia A. Turunen

**Affiliations:** 1 A.I. Virtanen Institute for Molecular Sciences, University of Eastern Finland, Kuopio, Finland; 2 RNatives Oy, Kuopio, Finland; 3 Department of Molecular and Experimental Medicine, The Scripps Research Institute, La Jolla, California, United States of America; 4 Wits/SAMRC Antiviral Gene Therapy Research Unit, School of Pathology, University of the Witwaterstrand, Witwaterstrand, South Africa; 5 Center for Gene Therapy, City of Hope–Beckman Research Institute at the City of Hope, Duarte, California, United States of America; 6 Menzies Health Institute Queensland, School of Medical Science Griffith University, Gold Coast Campus, Queensland, Australia; 7 Heart Center and Gene Therapy Unit, Kuopio University Hospital, Kuopio, Finland; 8 Department of Paediatrics, University of Oxford, Oxford, United Kingdom; 9 MDUK Oxford Neuromuscular Centre, Oxford, United Kingdom; Universitat des Saarlandes, GERMANY

## Abstract

MicroRNAs are well characterized in their role in silencing gene expression by targeting 3´-UTR of mRNAs in cytoplasm. However, recent studies have shown that miRNAs have a role in the regulation of genes in the nucleus, where they are abundantly located. We show here that in mouse endothelial cell line (C166), nuclear microRNA miR-466c participates in the regulation of vascular endothelial growth factor a (*Vegfa)* gene expression in hypoxia. Upregulation of *Vegfa* expression in response to hypoxia was significantly compromised after removal of miR-466c with CRISPR-Cas9 genomic deletion. We identified a promoter-associated long non-coding RNA on mouse *Vegfa* promoter and show that miR-466c directly binds to this transcript to modulate *Vegfa* expression. Collectively, these observations suggest that miR-466c regulates *Vegfa* gene transcription in the nucleus by targeting the promoter, and expands on our understanding of the role of miRNAs well beyond their canonical role.

## Introduction

MicroRNAs (miRNAs) are a class of small non-coding RNAs that typically repress genes by targeting the 3′-untranslated region (3′ UTR) of mRNAs via post-transcriptional gene silencing (PTGS), and can be considered endogenous triggers of RNA interference (RNAi) [1]. However, miRNA biology appears to be much more complex than traditionally thought, and RNAi machinery components have been found to be active in both the cytoplasm and nucleus [2]. We recently showed by small RNA-sequencing that miRNAs are abundant in the nucleus, that hypoxia induces changes in the miRNA levels both in the nucleus and cytoplasm, and that the majority of these changes occur preferentially in specific cellular compartments [3]. Recent studies have also identified nuclear roles for miRNAs [4]. Nuclear actions of small RNAs have been more extensively studied in other organisms such as plants, where early reports showed that non-coding RNAs were able to modulate DNA methylation [5]. However, recent studies in mammalian cells have suggested multiple novel modes of action for small RNAs, such as binding to long non-coding RNAs (lncRNAs) or regulating alternative splicing [4, 6]. For example, miR-9 has been shown to target a known lncRNA MALAT1 in the nucleus, leading to its degradation [7]. Small interfering RNAs (siRNAs) and double-stranded RNAs (dsRNAs) have been shown to regulate alternative splicing of mRNAs [8, 9] and similarly acting antisense oligonucleotides (ASOs) are already used clinically for exon skipping therapy [10]. To date, no studies on the regulation of alternative splicing by miRNAs have been published. However, this may likely be one of the nuclear roles of miRNAs, as splicing events occur quickly after transcription in the nucleus [11]. In addition, synthetic small RNAs and natural miRNAs have been shown to target gene promoters [12–17]. Interestingly, this promoter targeting can either repress or induce transcriptional gene expression [18]. It has been proposed that the targeted promoters express promoter-associated lncRNAs that are bound by the small RNAs. This recruits chromatin modifying factors to the site, inducing changes in the gene expression [19, 20]. Nonetheless, the ability to induce transcriptional gene activation (TGA) provides interesting therapeutic opportunities. We were the first to show this phenomenon occurs *in vivo* in a mouse model of hindlimb ischemia [18], and since that study, progress in therapeutic field has been made. TGA-based small activating RNA targeting C/EBP-α has been developed by MiNa Therapeutics [15] and was the first to enter phase I clinical trials for treatment of liver cancer [21].

Regulation of Vascular Endothelial Growth Factor A (VEGFA) levels is important for many gene therapy applications and has been under development for the treatment for conditions such as myocardial infarction and peripheral arterial disease. We have previously shown that TGA by shRNA targeting *Vegfa* promoter results in efficient treatment of hindlimb ischemia [18] and myocardial infarction [22]. Our sequencing study also identified a wide population of nuclear-localized endogenous miRNAs in endothelial cells [3]. Here we expand on our previous observations and investigate whether naturally occurring miRNAs endogenously target the *Vegfa* promoter and regulate its expression. We find that mmu-miR-466c targets the *Vegfa* promoter to regulate its expression through interactions with a promoter lncRNA, and that this interaction is important for *Vegfa* expression in hypoxia. Our observations suggest that in addition to artificial small RNAs, endogenous miRNAs are capable of also targeting and regulating gene promoters. Further, we find that these endogenous miRNAs function to upregulate gene transcription in the nucleus, as we show here with endogenous miR-466c.

## Results

### miR-466c is predicted to target the *Vegfa* promoter and is induced in the nucleus by hypoxia

**Fig 1:**
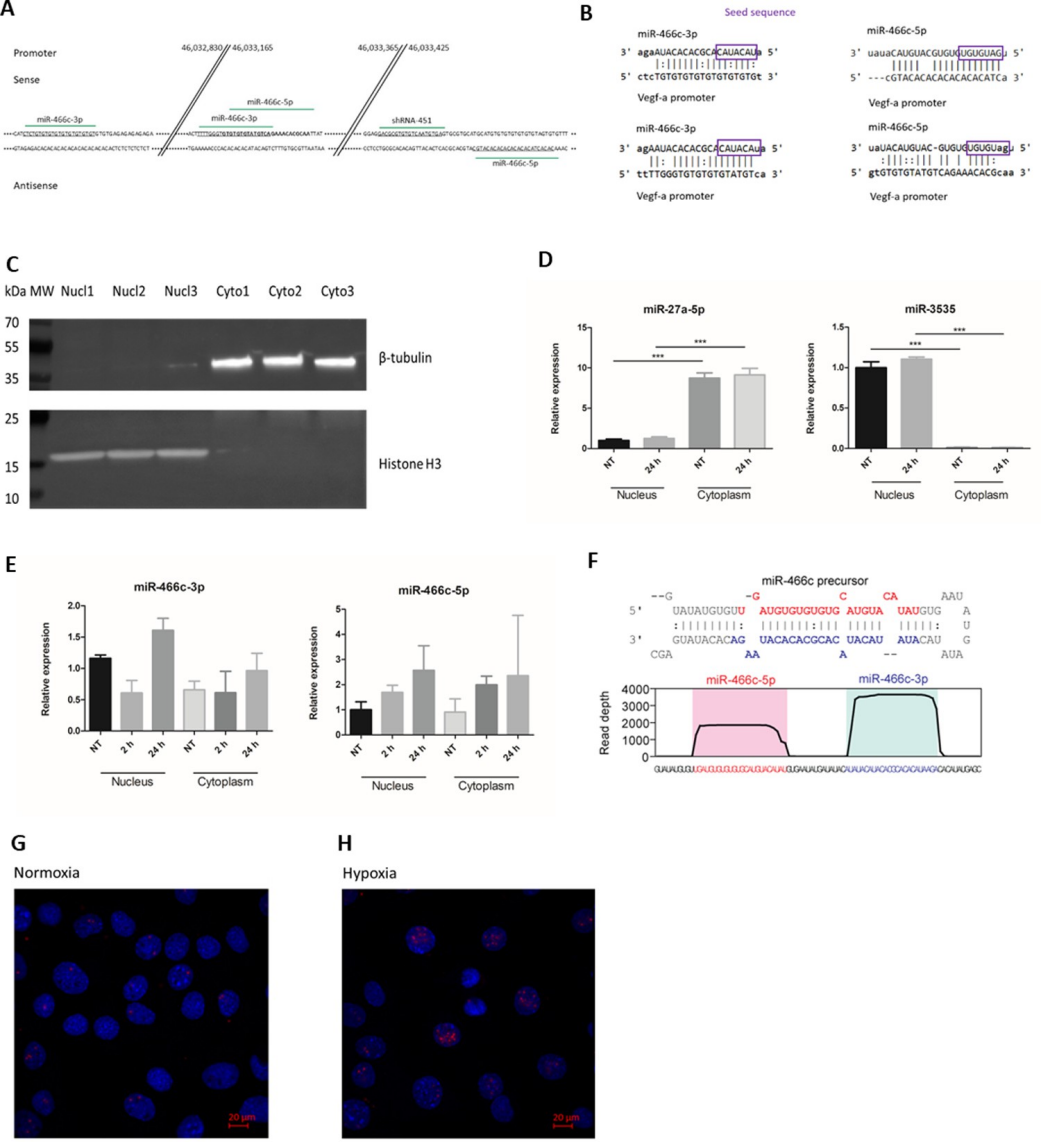
mmu-miR-466c is predicted to bind *Vegfa* gene promoter and is found in the nucleus of endothelial cells. A. Schematic figure showing the predicted targeted loci of mmu-miR-466c on *Vegfa* promoter. Numbers refer to locus on chromosome 17. shRNA-451 is published previously for targeting *Vegfa* promoter and upregulating its expression [18]. miR-466c targets were predicted using RegRNA 2.0. B. Sequence alignment showing miR-466c binding to promoter transcripts. Seed sequence of miR-466c is marked with purple box. C. Mouse endothelial cells (C166 cell line) were fractionated to nuclear and cytoplasmic fractions to study the compartmentalization of miRNAs. Purity of fractions was verified with western blot against cytoplasmic and nuclear markers β-tubulin and histone H3, respectively. n = 3. D. Purity of fractions was also verified by qPCR of cytoplasmic and nuclear miRNAs miR-27a-5p and miR-3535, respectively. NT is normoxic control sample, 24h is sample treated with hypoxia for 24h. n = 3, ANOVA with Bonferroni correction was used for determining the statistical significance, data is represented as mean ± SD. E. Both miR-466c-3p and miR-466c-5p were detected from both nucleus and cytoplasm of C166 cells by RT-qPCR. miR-466c expression was increased upon hypoxia, especially miR-466c-3p was induced in the nuclear fraction of the cells. NT is normoxic control sample, 2h and 24h are samples treated with hypoxia for 2h or 24h, respectively. n = 3–4, data is represented as mean ± SD. F. miR-466c precursor structure, from which both miR-466c-5p and miR-466c-3p are processed. miRBase graph shows how deep sequencing reads of the mature miRNAs are distributed and miR-466c-3p is the more abundant mature miRNA. G. miRNA-FISH for miR-466c-3p confirms the nuclear localization of the mature miRNA both in normoxia and hypoxia (24h timepoint). *P ≤ 0.05, **P ≤0.01, ***P ≤ 0.001.

We first performed a bioinformatic screen to identify putative miRNA target sites contained within the murine *Vegfa* promoter. A 700 bp fragment of DNA upstream of the annotated *Vegfa* transcription start site was retrieved and searched for target sites in both sense and antisense orientations using RegRNA 2.0 [23], which itself uses the miRanda algorithm [24] for target site prediction. Multiple members of miR-466c family were predicted to target *Vegfa* promoter (Fig 1A and 1B). Interestingly, both miR-466c-5p and miR-466c-3p were predicted to target sites on the promoter near sites previously described to be susceptible to shRNA mediated TGA [18] (Fig 1A). To analyze the expression of miR-466c in our experimental setting, we next cultured mouse endothelial C166 cells in normoxia or hypoxia and collected samples at 2h or 24h for nuclear-cytoplasmic fractionation followed with RNA and protein isolation. Successful separation of nuclear and cytoplasmic fractions was confirmed by western blot for histone H3 (nuclear marker) and β-tubulin (cytoplasmic marker) (Figs 1C and S1), and RT-qPCR for miR-3535 (nuclear) and miR-27a (cytoplasmic), consistent with our previous study [3] (Fig 1D). The expression of miR-466c, both 3p and 5p arms, was found to be increased upon hypoxia in C166 endothelial cells as analyzed by RT-qPCR (Fig 1E). The 3p arm was observed to be the most abundant form, both in nucleus and in the cytoplasm. This strand bias was also observed in publicly available small RNA-seq data taken from miRbase [25] (Fig 1F). We used RNA *in situ* hybridization (miRNA FISH) to visualize the localization of miR-466c-3p in normoxic and hypoxic C166 cells (Fig 1G and 1H). With miRNA FISH, we verified that miR-466c is observed in the nucleus both in normoxia and hypoxia.

### miR-466c regulates *Vegfa* expression in endothelial cells

**Fig 2:**
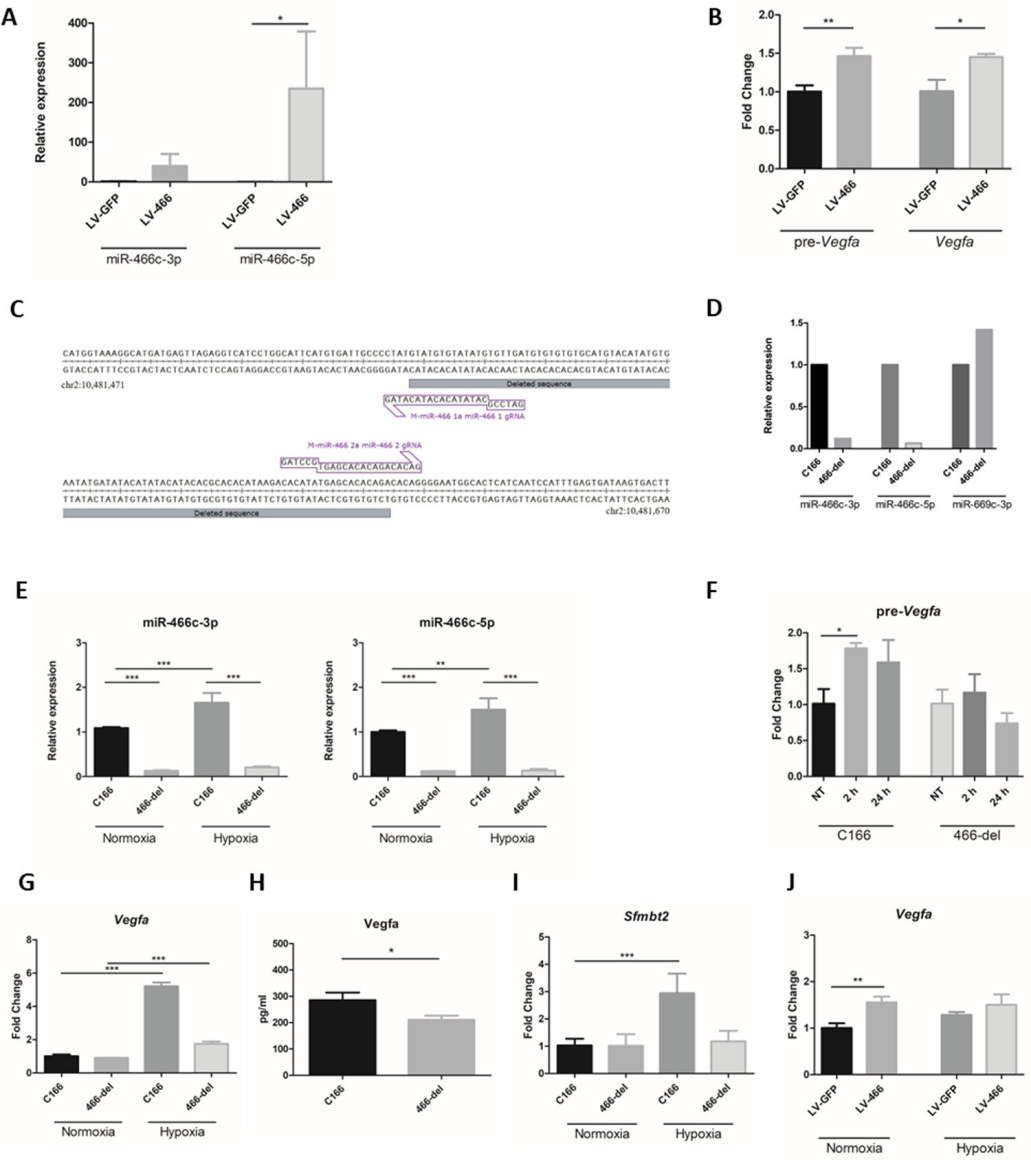
miR-466c participates in the regulation of *Vegfa* expression. A. Lentiviral expression of miR-466c increased both miR-466c-3p and miR-466c-5p levels in C166 cells. Cells were transduced with MOI 10 and samples collected 3d after transduction. C166 cells transduced with lentiviral vector expressing GFP only (LV-GFP) were used as control. n = 3, ANOVA with Bonferroni correction was used for determining the statistical significance, data is represented as mean ± SD. B. Overexpression of miR-466c (LV-466) in C166 cells resulted in increased transcription of pre-*Vegfa* and mature *Vegfa* as compared to control GFP only vector (LV-GFP), using RT-qPCR. n = 3, t-test was used for determining the statistical significance, data is represented as mean ± SD. C.To study the effects of knockdown of miR-466c, CRISPR-Cas9-mediated deletion of miR-466 from the C166 genome was performed. Schematic figure illustrates the deletion position in Sfmbt2 gene intron 10 in chromosome 2. D. Deletion of miR-466c resulted in a significant drop in miR-466c levels in the deletion cell line. qPCR for miR-669c, a miRNA expressed from the same cluster as miR-466c from Sfmbt2 gene intron 10, showed that CRISPR-Cas9-deletion of miR-466 precursor does not decrease the levels of miR-669c in the cells, whereas both strands of miR-466c are repressed. n = 1 E. Deletion of miR-466c resulted in a significant drop in miR-466c levels in the 466-del deletion cell line compared to parental C166 cells, and hypoxia did not induce miR-466c levels. n = 4, ANOVA with Bonferroni correction was used for determining the statistical significance, data is represented as mean ± SD. F. pre-*Vegfa* expression increases upon hypoxia in C166 cell line but does not change upon hypoxia in 466-del cell line. n = 3, ANOVA with Bonferroni correction was used for determining the statistical significance, data is represented as mean ± SD. *G*. *Vegfa* gene expression increases in hypoxic conditions in parental C166 cell line, but not to same extent in 466-del cell line. n = 4, ANOVA with Bonferroni correction was used for determining the statistical significance, data is represented as mean ± SD. H. Vegfa protein expression is decreased in 466-del cell line compared to parental C166 cells. n = 3, t-test was used for determining the statistical significance, data is represented as mean ± SD. I. Parental gene *Sfmbt2* expression is upregulated upon hypoxia in parental C166 cells, but not in miR-466-deletion cell line 466-del. n = 4, ANOVA with Bonferroni correction was used for determining the statistical significance, data is represented as mean ± SD. J. Overexpression of miR-466c with lentiviral vector in miR-466c-deletion cell line does not return the hypoxia responsiveness to parental cell level. n = 4, t-test was used for determining the statistical significance, data is represented as mean ± SD. *P ≤ 0.05, **P ≤0.01, ***P ≤ 0.001.

We next sought to see if manipulation of miR-466c levels in C166 cells would lead to changes in *Vegfa* expression using lentiviral vector overexpression system (LV-466). Transduction of C166 cells resulted in elevated levels of both miR-466c-3p and miR-466c-5p (Fig 2A). We analyzed the expression of both pre-*Vegfa* and mature mRNA *Vegfa* from cells transduced with LV-466 to determine any changes in transcription. Overexpression of miR-466c increased both pre-*Vegfa* and mature *Vegfa* levels 1.5-fold in these cells, which is consistent with previous observations with *Vegfa* upregulating shRNA [18]. These data indicate that miR-466c over-expression functionally activates *Vegfa* gene transcription (Fig 2B).

### Removal of miR-466c significantly reduces *Vegfa* expression in hypoxic endothelial cells

To analyze the role of miR-466c in the regulation of *Vegfa* in hypoxic response, we used CRISPR-mediated gene editing to remove 97 bp region from the *Sfmbt2* intron 10 which contains the miR-466c hairpin from the parental C166 cell line (Fig 2C). Clonal cell populations were generated and screened for miR-466c expression, as well as miR-669c-3p, which is expressed from the same miRNA cluster in *Sfmbt2* intron 10, expression. *Sfmbt2* intron cluster contains two other miR-466c pre-miRNA hairpins, but removal of this one copy (mmu-mir-466c-1) was sufficient to dramatically reduce miR-466c expression, whereas miR-669c-3p expression was not changed, indicating that the CRISPR removal of miR-466c was not affecting the other miRNAs in this cluster (Fig 2D). After removal of miR-466c (466-del cell line), the hypoxia-mediated induction of miR-466c was lost (Fig 2E). *Vegfa* expression is known to be upregulated upon hypoxia [26]. Importantly, when miR-466c was removed, the upregulation of *Vegfa* expression in response to hypoxia was also diminished as determined by RT-qPCR of pre-*Vegfa* (Fig 2F) and mature *Vegfa* mRNA (Fig 2G). In addition, Vegfa protein expression was decreased after removal of miR-466c (Fig 2H). The expression of the parent gene, *Sfmbt2*, was also not found to be induced by hypoxic stimuli (Fig 2I). Rescue of miR-466c expression in the 466-del cell line via lentiviral overexpression was sufficient to increase *Vegfa* levels both in normoxic and hypoxic conditions, but hypoxia did not further induce *Vegfa* expression to the normal extent (Fig 2J).

**Fig 3:**
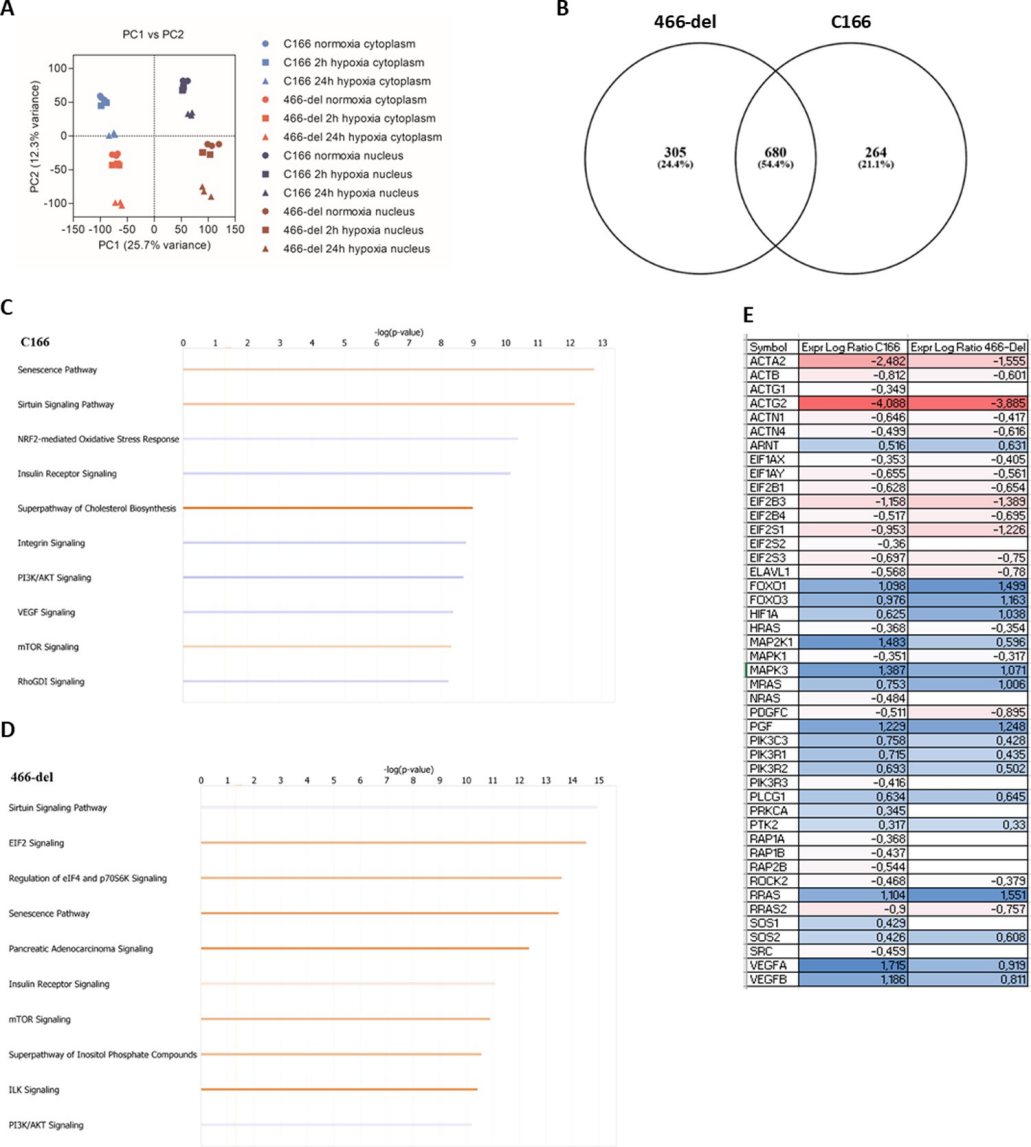
Total transcriptome sequencing of parental and miR-466c deletion cell lines shows significant differences on gene expression profiles. A. Both C166 and 466-del miR-466c deletion cell lines were subjected to hypoxia (2h or 24h), fractionated to nuclear and cytoplasmic samples and analyzed by RNA-sequencing. PCA plot of all sequenced groups shows that the samples are grouped by difference in nuclear and cytoplasmic transcripts, but also by cell line. B. Venn diagram of differentially expressed genes in parental C166 and 466-del deletion cell line shows that most changes in gene expression (680 differentially expressed genes) are still shared in both cell lines. C. Comparison analysis of 24h hypoxia vs. normoxia in cytoplasm of parental C166 cell line. Top 10 canonical pathways altered are shown in the figure, red colour indicates positive z-score, blue indicates negative z-score. D. Comparison analysis of 24h hypoxia vs. normoxia in cytoplasm of 466-deletion cell line. Top 10 canonical pathways altered are shown in the figure, red colour indicates positive z-score, blue indicates negative z-score. E. VEGF signalling pathway members were differentially regulated in C166 and 466-deletion cell line in same fashion, but to different extent.

To further analyze the changes in global gene expression, we performed whole transcriptomic sequencing from nuclear and cytoplasmic fractions of C166 and 466-del cell lines grown in normoxia and hypoxia. As depicted by PCA plot, gene expression profiles between the two cell lines differ remarkably both in cytoplasm and nucleus (Fig 3A). When looking at all differentially expressed genes in the cell lines, we observe that majority of the genes differentially regulated in the cell lines are shared (680 genes), whereas the mutation induces changes in expression of 305 genes that are not deregulated in parental cell line, and 264 genes are not differentially expressed in 466-del cell line even though they are found in the parental line (Fig 3B). We analyzed the changes more closely using ingenuity pathway analysis (IPA). Many pathways where changes were associated were shared between the cell lines (sirtuin signalling, senescence, insulin receptor signalling, PI3K/AKT signalling, mTOR signalling), but some pathways were different (e.g. EIF2 signalling in mutation cell line and NRF2-mediate oxidative stress response in parental cell line) (Fig 3C and 3D). We also analyzed the individual members of VEGF signalling pathway, and noticed that most of the genes were still regulated in similar fashion after miR-466 removal (e.g. downregulated in both cell lines), but the extent of regulation was notably different (Fig 3E). The most differentially regulated VEGF signalling pathway member was MAP2K1, which was significantly downregulated following miR-466 deletion.

### Identification of a non-coding transcript on the mouse *Vegfa* promoter

**Fig 4:**
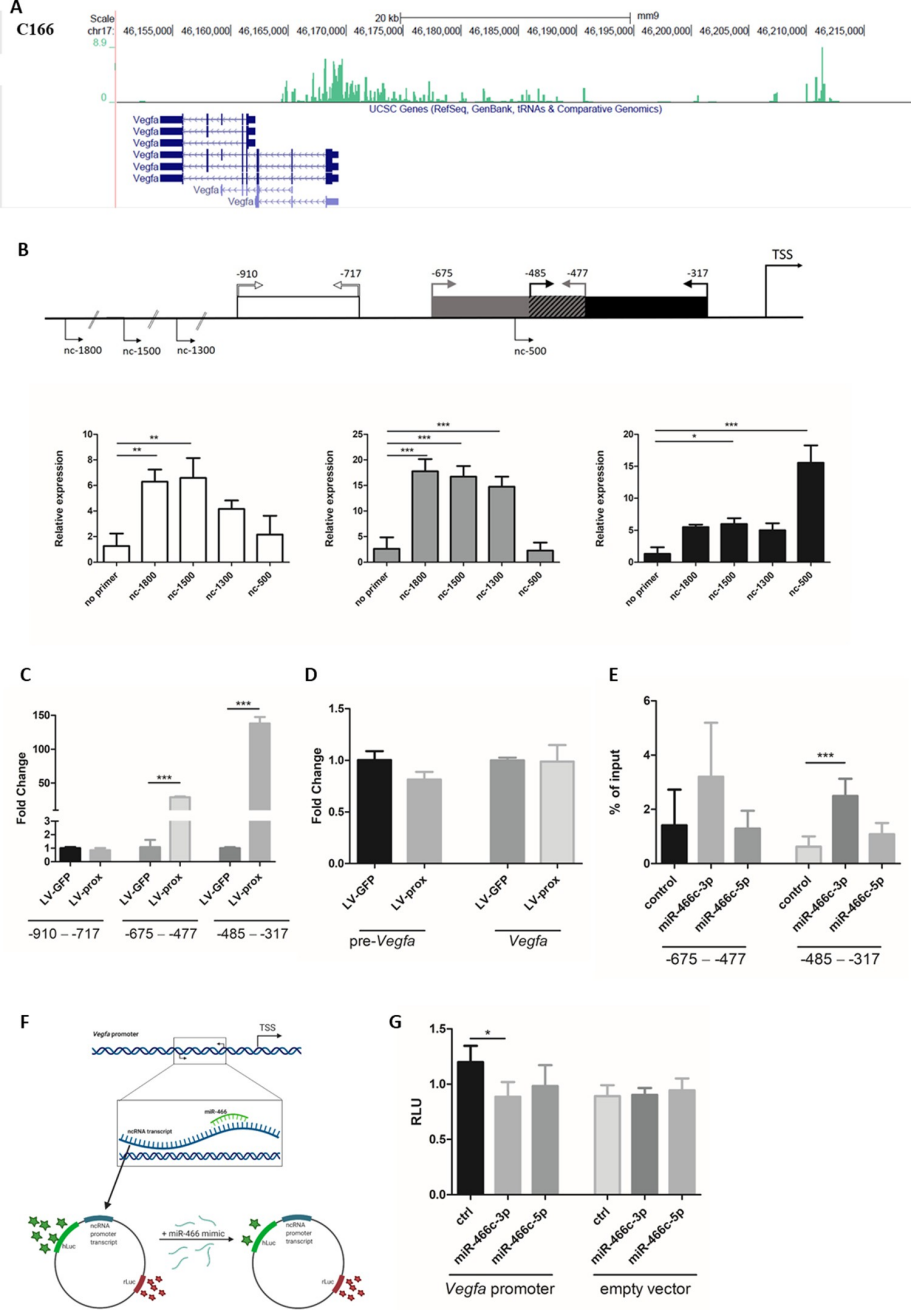
Murine *Vegfa* promoter is associated with antisense non-coding transcript, which is the target for miR-466c. A. GRO-seq shows antisense lncRNA at *Vegfa* promoter in C166 cells. B. Antisense ncRNA is detected from *Vegfa* promoter using strand-specific qPCR. ncRNA is found from the regions that were predicted for miR-466c targeting. n = 3, ANOVA with Bonferroni correction was used for determining the statistical significance, data is represented as mean ± SD. C. qPCR of ncRNA shows increased levels of antisense ncRNA after transduction with lentiviral vector encoding the proximal promoter region (LV-prox). n = 3, t-test was used for determining the statistical significance, data is represented as mean ± SD. D. Overexpression of *Vegfa* promoter-associated ncRNA with lentiviral vector (LV-prox) does not affect expression levels of *Vegfa*. n = 3, data is represented as mean ± SD. E. Biotinylated miR-466c mimic was used to pulldown RNA transcripts binding to miR-466c. *Vegfa* promoter associated ncRNA was detected from the pulldown samples by RT-qPCR. n = 5, ANOVA with Bonferroni correction was used for determining the statistical significance, data is represented as mean ± SD. F. Schematic illustration of luciferase assay design. Figure was created with BioRender.com. Republished from BioRender.com under a CC BY license, with permission from BioRender.com, original copyright 2022. G. Direct interaction between miR-466c and *Vegfa* promoter-associated ncRNA was confirmed using luciferase assay, where miR-466c-3p transfection resulted in significant regulation compared to control. Empty luciferase vector showed no difference with any of the transfected mimics. n = 4, t-test was used for determining the statistical significance, data is represented as mean ± SD. *P ≤ 0.05, **P ≤0.01, ***P ≤ 0.001.

Previous studies demonstrated that transcriptional gene silencing (TGS) and transcriptional gene activation (TGA) may mechanistically require bidirectional transcription and/or promoter associated transcripts [16, 20, 27]. Promoter-associated transcripts for mouse *Vegfa* have not been previously characterized. Therefore, we performed Global Run-On-sequencing (GRO-seq) in order to determine if there is bidirectional transcription at the *Vegfa* promoter. Notably, the promoter was found to have an antisense transcript located up to ~27 kb upstream from its reported transcription start site (Fig 4A). Utilizing publicly available GRO-seq data, we can see that the promoter of *Vegfa* is transcribed also in other murine cell lines (3T3L1, AtT20, B-cell, Liver, MEF, Muscle, Neuron) (S2 Fig). For RT-qPCR confirmation of the transcript, cDNA was synthesized using antisense directional primers located at different promoter loci (nc-1800, nc-1500, nc-1300 and nc-500, where the number indicates the distance upstream of *Vegfa* TSS). qPCR was then performed with primer pairs at three different loci (-910 − -717, -675 − -477 and -485 − -317, relative to TSS) and confirmed the presence of antisense transcript on the proximal promoter of *Vegfa* (Fig 4B). These observations suggest that the promoter targeted shRNAs identified previously [18] and the miRNAs identified here may exert their action on the *Vegfa* promoter via interactions with this antisense transcript. The promoter sequence of *Vegfa* was further cloned into LV-vector, and the expression of the promoter non-coding RNA produced by the lentivirus was confirmed by RT-qPCR (Fig 4C). Interestingly, transduction of this non-coding RNA to C166 cells did not significantly affect *Vegfa* expression (Fig 4D). These data suggest that the ncRNAs may require the correct chromosomal context.

To show the miR-466c targeting of promoter-associated transcript on *Vegfa* promoter, we performed biotin pulldown assay using biotinylated miR-466c mimics transfected to C166 cells. Since the promoter transcript was detected at very low levels in RT-qPCR, we first increased its expression by lentiviral vector (LV-prox). RT-qPCR analysis showed that miR-466c-3p binds the proximal promoter transcript, and we detect the pulled down transcript at locus -485 − -317 and -675 − -477 bp upstream from TSS (Fig 4E), consistent to the bidirectional RT-qPCR analysis of LV-prox induced transcript (Fig 4B). We also performed luciferase reporter assay to analyze the binding of miR-466c to the promoter transcript. Promoter sequence (S1 Table) was cloned into luciferase reporter plasmid and transfected to HEK293T cells together with miR-466c-3p, miR-466c-5p or control mimic. miR-466c-3p showed significant reduction in luciferase activity compared to control and is therefore able to bind the transcript on *Vegfa* promoter.

## Discussion

Previous studies have shown that, in addition to well described role in silencing of gene expression by targeting 3´-UTR of mRNAs in cytoplasm (post-transcriptional gene silencing, PTGS), miRNAs have a role in the regulation of gene expression in the nucleus by TGS and TGA. We showed recently that vast number of miRNAs are preferentially located in the nuclear fraction of endothelial cells and that their distribution between cytoplasm and nucleus is affected by stimulus such as hypoxia [3]. Non-canonical roles of miRNAs are still largely unknown and less studied than their action in cytoplasmic RNAi. However, since miRNAs are abundantly present as mature miRNAs in the nucleus, it thus seems likely that they participate in nuclear functions.

We have previously demonstrated regulation of *Vegfa* expression by transducing cells with lentiviral vectors encoding promoter-targeted shRNAs. Depending on their target site, shRNAs are able to upregulate (TGA) or downregulate (TGS) *Vegfa* expression [18]. The shRNAs were shown to have efficient therapeutic function both in hindlimb ischemia and myocardial infarction models [18, 22]. These observations suggested that shRNAs may function by mimicking structurally similar endogenous miRNAs that could act in a same manner *in vivo* and potentially regulate *Vegfa* expression by targeting *Vegfa* promoter. In the study presented here, such nuclear miRNA with target sites in the murine *Vegfa* promoter was identified. Some miR-466c predicted targets were close to the previously described site that is subjectable to TGA by shRNA [18]. The shRNA was designed to be fully complementary to the promoter sequence, whereas for endogenous miRNAs, binding is more partial although the binding of seed sequence is considered often critical. Our analysis shows that miR-466c exhibits partial complementarity, also from other parts than seed sequence, to the *Vegfa* promoter at several sites.

As seen from miRBase, miR-466c-3p is more abundantly expressed across studies. However, miR-466c-5p, although expressed at lower levels, is found both in our analysis by RT-qPCR in C166 cells, as well as in other studies by sequencing, as represented by miRBase data. The processing of miRNAs and miRNA strand selection may occur differently in different cell types or conditions. In many cases, processing of miRNA leads to one functional “active strand” which is incorporated into RISC and the other “passenger strand” is degraded. However, both strands of miRNA may be equivalently functional and participate in gene regulation, possibly by coordinated pathways [28]. We speculate that this may also be the case with miR-466c in hypoxic endothelial cells, where both strands could have a regulatory and compensatory role in the regulation of *Vegfa*. This might even occur by targeting both promoter via TGA and mRNA 3ʹUTR via traditional PTGS and thus creating a regulatory network loop to fine-tune the expression of essential genes.

The miR-466c precursor resides within intron 10 of the polycomb gene *Sfmbt2* [29]. *Vegfa* is known to be regulated in response to hypoxia and the observations presented here demonstrate that *Sfmbt2* expression is similarly increased upon hypoxia, as does the expression of miR-466c. Overexpression of miR-466c using lentiviral vector (LV-466) increased pre- and mature mRNA levels of *Vegfa*. *Vegfa* is regulated on multiple levels post-transcriptionally, including increased stability of mRNA upon hypoxia [30], but our result clearly indicates increased transcription of the gene in case of overexpression of miR-466c, thereby indicating potential TGA by miR-466c. Removal of miR-466c by CRISPR decreased Vegfa protein levels but did not induce any changes in *Vegfa* mRNA expression at basal levels, but this may be due to the longer timepoint (4d) used in protein experiments and accumulation of Vegfa protein to the cell culture medium. However, deletion of miR-466c impaired the upregulation of *Vegfa* typically observed following hypoxic stimulus. It is possible that other compensatory mechanisms regulate basal level expression of *Vegfa*, but miR-466c is essential for the induction of gene expression followed by hypoxic stimulus. When miR-466c was returned to the miR-466c-deleted cell line by lentiviral overexpression, we detect upregulation in *Vegfa* expression already at normoxia, corresponding to the overexpression experiment in parental intact C166 cells. However, complete restoration of hypoxia-induced upregulation does not occur in the absence of endogenous miR-466c.

Previous research has shown that TGS and TGA often require the presence of non-coding transcripts on the targeted promoter [20]. Studies on the human *VEGFA* promoter have identified promoter-associated antisense and sense transcripts which would be bound by promoter targeted small RNAs [31, 32]. We performed GRO-seq and located an antisense RNA transcript on the mouse *Vegfa* promoter in C166 endothelial cells and confirmed it by directional RT-qPCR. This promoter-associated RNA transcript is most likely the target of the miRNAs and shRNAs found to target and regulate *Vegfa* promoter and affect hypoxia induced modulation of *Vegfa*. This was verified with biotin pulldown and luciferase reporter assays, which both showed miR-466c to target and directly bind the promoter transcript. It is possible that the target abundance determines the localization of miRNA, as has been observed with cells transfected with siRNA that selectively shuttle to those compartments containing the small RNA target [33]. Importin-8 has been shown to transport the mature miRNAs from cytoplasm to the nucleus together with Ago2 [34]. Sequencing experiments have shown that miRNA abundance overlaps between nucleus and cytoplasm, suggesting that nuclear miRNAs are shuttled to and from the cytoplasm [2, 35].

**Fig 5:**
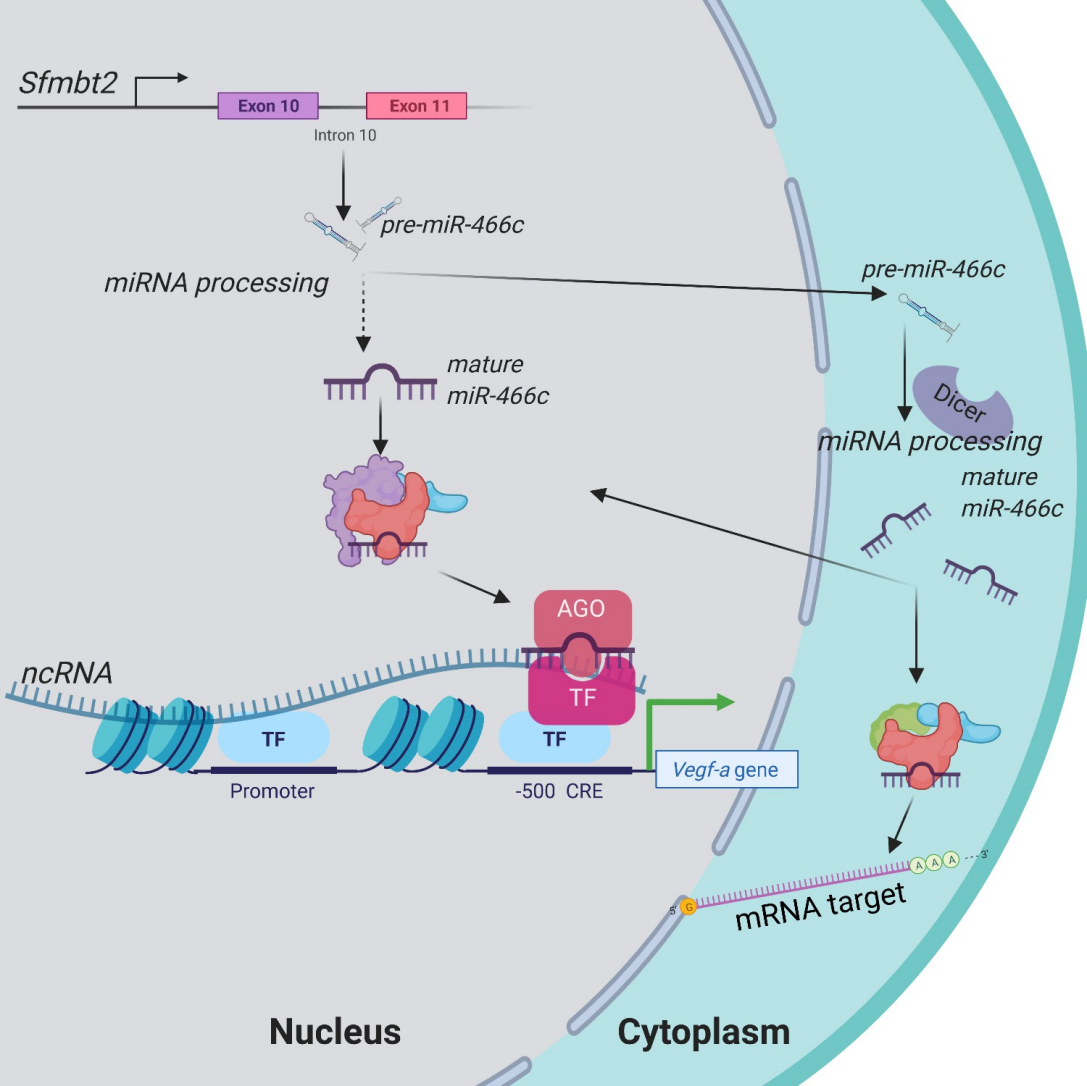
Schematic illustration depicting miR-466c targeting of murine *Vegfa* promoter.

The location in the promoter where shRNA was designed to target and where miR-466c was predicted to target *Vegfa* is a “hot spot” for *Vegfa* activation and contains Hif1α and Yy1 binding sites and a cAMP response element (CRE). It is well known that hypoxia induces Hif1α and Yy1 expression [36–38]. Both of these transcription factors have predicted target loci in the *Sfmbt2* promoter as well and therefore could be involved in the regulation of *Sfmbt2* induction in hypoxia, which as shown here results in increased miR-466c expression. It is likely that pre-miRNAs expressed from the genome are processed in multiple steps both in nucleus (Drosha/DGCR8) and in cytoplasm (Dicer) [39] and some mature miRNAs are directed and shuttled back to nucleus. However, some publications have shown the existence of nuclear Dicer and RNAi factors, so it is possible that nuclear miRNAs are processed completely in the nucleus [2, 40]. *Vegfa* promoter harbours a ncRNA transcript, which can be bound by miR-466c to recruit TGA-associated chromatin modifying proteins to the site [18]. We have previously shown that preventing CBP-CREB interaction inhibited the shRNA-mediated Vegfa activation [22], which is likely due to the conserved CRE [41] located at the shRNA target site. Also, the predicted target sites of miR-466c on the *Vegfa* promoter are in proximity to the CRE and therefore the molecular mechanism of miR-466c could be similar to the action of the shRNA. A putative model for the *Vegfa* regulation by miR-466c in hypoxia based on these observations is presented in Fig 5.

Collectively, the observations presented here regarding the nuclear functions of miR-466c, along with several previous reports, suggest that small non-coding RNAs, both endogenously and exogenously expressed, modulate gene activation in the nucleus by a naturally occurring biological mechanism that also involves interactions with promoter associated long ncRNAs. This hypoxia induced ncRNA network of transcriptional regulation significantly expands our understanding of the role of miRNAs well beyond their canonical role in regulating PTGS and ultimately suggests that a vibrant world of ncRNAs and regulatory potential resides in the nucleus.

miR-466c is expressed from *Sfmbt2* intron 10 and processed to mature miRNAs, miR-466c-3p and miR-466c-5p. Mature miRNAs are transported back to the nucleus, where they re-locate to their target sites in non-coding RNA on *Vegfa* gene promoter. Binding of miR-466c to the promoter-associated ncRNA recruits transcription factors and epigenetic modifiers to the chromatin locus, thus resulting in upregulation of *Vegfa*. Figure was created with *BioRender*.*com*. Republished from BioRender.com under a CC BY license, with permission from BioRender.com, original copyright 2022.

## Materials and methods

### Cell culture

C166 (yolk-sac derived mouse endothelial cell line, ATCC:CRL-2581), 466-del and HEK293T (ATCC: CRL-11268) cells were maintained under normal conditions (37°C, 5% CO_2_) in Dulbecco’s Modified Eagle’s Medium (DMEM) (Sigma-Aldrich, Germany) containing 10% fetal bovine serum (FBS) (Thermo Fisher Scientific, USA) and 1% penicillin-streptomycin (PS) (Thermo Fisher Scientific). In hypoxia experiments, cells were cultured for 2h or 24h in a hypoxia chamber with 1% O_2_, 5% CO_2_ (InvivO2, Baker Ruskinn, UK). The nuclear-cytoplasmic fractioning of the cells was performed as described previously [42].

### miRNA target predictions

Mouse *Vegfa* promoter sequence encompassing 700 bp of upstream promoter sequence of the *Vegfa* gene was retrieved from Genome Browser [43] (https://genome.ucsc.edu/; Mouse genome Dec. 2011 (GRCm38/mm10). Promoter sequence was submitted to RegRNA 2.0 [23] (http://regrna2.mbc.nctu.edu.tw/detection.html) tool to predict targeting miRNAs.

### Western blot

Protein samples were extracted from nuclear and cytoplasmic fractions according to protocol by Gagnon et al. [42] and with TRI reagent (Sigma-Aldrich) according to the manufacturer’s instructions. For western blot, equal volumes of extracted protein were loaded on precast gels (Mini-Protean TGX Precast Gel, 4–15%, Bio-Rad, USA) and transferred to nitrocellulose membranes (Trans-Blot Turbo Midi 0.2 μm Nitrocellulose Transfer Packs, cat. #1704159, Bio-Rad). The membranes were blocked with 5% milk for 1 hour at room temperature, washed with TBST (0.15 M sodium chloride, 0.050 M TRIS-HCl buffer; 0.05% Tween 20; pH 7.6) and incubated with antibodies against a known nuclear protein (anti-trimethyl-histone H3 (Lys27), 1:2,500 dilution, cat. 07–449, Merck, Germany) and a cytoplasmic protein (anti-β-tubulin, 1:1,000 dilution, cat. T5201, Sigma-Aldrich) overnight at 4°C. The membranes were washed with TBST and incubated with secondary antibodies (alexa fluor 488 goat anti-rabbit IgG (H + L), 1:3,000 dilution, cat. A11034, Invitrogen by Thermo Fisher Scientific; alexa fluor 488 goat anti-mouse IgG, 1:2,500 dilution, cat. A11001, Invitrogen by Thermo Fisher Scientific) for 1 hour at room temperature. Membranes were washed with TBST and imaged with the ChemiDoc MP Imaging System (Bio-Rad).

### RNA extraction, cDNA synthesis and RT-qPCR

RNA was extracted from nuclear and cytoplasmic fractions according to protocol by Gagnon et al. [42] using TRI reagent (Sigma-Aldrich). Total cellular RNA was extracted with TRI reagent (Sigma Aldrich) according to the manufacturer’s instructions and treated with DNase I, RNase-free (Thermo Fisher Scientific). For miRNA analysis, cDNA synthesis was performed using the TaqMan MicroRNA Reverse Transcription Kit (Applied Biosystems by Thermo Fisher Scientific) according to manufacturer’s protocol and analyzed with qPCR using miRNA-specific TaqMan assay (mmu-miR-466c-3p ID: 464896_mat; mmu-miR-466c-5p ID: 463771_mat, mmu-miR-669c-3p ID: 464620_mat, hsa-miR-27a-5p ID: 002445; mmu-miR-3535 ID: CTEPR23; Thermo Fisher Scientific). For gene expression analysis, cDNA was synthesized using RevertAid Reverse Transcriptase (Thermo Fisher Scientific) and random hexamer primer (Thermo Fisher Scientific). In experiments detecting non-coding RNA transcript on *Vegfa* promoter, specific primers (nc-500, nc-1300, nc-1500 and nc-1800; listed in S1 Table, all from Integrated DNA Technologies IDT, USA) were used in cDNA synthesis. cDNA quantification was performed using TaqMan Gene Expression Assays (*Vegfa* ID: Mm00437306_m1; *Sfmbt2* ID: Mm00616783_m1; endogenous control *Gapdh* ID: Mm99999915_g1; *Actb* ID: Mm00607939_s1, Thermo Fisher Scientific) and custom designed primers (pre*-mVegfa* and *Vegfa* promoter primers, Integrated DNA Technologies IDT) (S1 Table). Samples were quantified by using Maxima Probe/ROX qPCR Master Mix (2X) (Thermo Fisher Scientific). Thermal cycling was performed using a LightCycler® 480 Instrument II (Roche, Switzerland) with the following program: 10 min at 95°C, followed by 50 cycles of 15 s at 95°C and 60 s at 60°C. miRNA RT-qPCR was started with an additional step of 2 min at 50°C. RT-qPCR data were analyzed using the ΔΔCq method where normalization was available, or the ΔCq method for un-normalized data.

### miRNA fluorescent *in situ* hybridization (FISH)

For miRNA FISH, C166 cells were seeded on 8-well chamber slides and grown in normoxia or hypoxia (24h). ViewRNA miRNA ISH Cell Assay Kit (Thermo Fisher Scientific) was used for the hybridizations according to the manufacturer’s protocol. ViewRNA Cell Plus Probe Set (Affymetrix/Thermo Fisher Scientific) was used to detect mmu-miR-466c-3p (miR-466p-3p, assay ID: VM1-21572, detection label Alexa Fluor 546). Cell nuclei were visualized using DAPI stain. Pictures were taken using ZEISS LSM700 confocal microscope using 40× oil objective and analyzed with ZEN lite blue 2.6 software (ZEISS, Germany).

### Lentiviral transductions

For overexpression of miR-466c, 255 bp locus of genomic sequence from Sfmbt2 intron 10, which contains the miR-466c hairpin (S1 Table), was cloned into third generation human immunodeficiency virus 1 (HIV-1)–based LV-PGK-GFP-U6-RNA vector (LV-466). For overexpression of *Vegfa* promoter ncRNA, genomic sequence from *Vegfa* promoter region between -671 − -115 bp relative to TSS was cloned into the lentiviral vector (S1 Table). As a control, we used lentivirus (LV) encoding only GFP (LV-GFP). The vectors were prepared by standard calcium phosphate transfection method in 293T cells [44]. C166 cells were transduced with lentiviral vector expressing either mmu-miR-466c (LV-466) or proximal non-coding RNA (LV-prox) using MOI 10 and samples were collected 3 days after the transduction (LV-466) or after three weeks of culturing (LV-prox).

### Deletion of miR-466c with CRISPR

In order to remove miR-466c from intron 10 of *Sfmbt2*, two guide RNAs were cloned into separate expression plasmids (pcDNA-H1-sgRNA) (S1 Table) and transfected into C166 cells along with Cas9 plasmid co-expressing GFP (PX458, Addgene, USA) using Nucleofector I (Amaxa/Lonza Bioscience, Germany). Based on GFP positivity, single cells were sorted into 96-well plate wells using sorting FACS (BD FACSARIA III Cell Sorter, BD Biosciences, USA) and clonal cell populations established. Cultures were genotyped by PCR (primers listed in S1 Table) to identify cells that contained the desired deletion, and positive clones further confirmed by Sanger sequencing.

### Enzyme-linked immunosorbent assay (ELISA)

C166 and 466-del cells were split to 6-wells (30 000 cells/1 ml/6-well; three replicates each cell line) in no phenol red DMEM with 10% FBS and 1% PS. After four days, medium samples were collected and centrifuged at +4 C 18 000 g for 20 min. Supernatants were discarded and samples resuspended to 100 ul NP-40 buffer (150 mM NaCl, 0,1% Triton X-100, 50 mM Tris-HCl, 1x protease inhibitor). Total protein amount of the samples was measured using BCA protein assay kit (Thermo Fisher Scientific) according to manufacturer’s protocol. 10 ug total protein was used for mVegf ELISA assay (Quantikine ELISA, mouse VEGF, Cat. No.: MMV00) according to manufacturer’s protocol. Victor^2^ WALLAC 1420 Multilabel counter (PerkinElmer) was used to measure wavelength 450nm and background wavelength 544nm was subtracted from those values.

### Whole transciptome sequencing and analysis

Parental C166 cells or miR-466c-deletion cell line (466-del) were cultured in normoxia or hypoxia for 24h. Cells were fractionated to nuclear and cytoplasmic fractions and RNA was isolated as described earlier. Whole transcriptome sequencing was performed as a service from Exiqon A/S (Vedbaek, Denmark). The library preparation was done using TruSeq® stranded total RNA sample preparation kit with rRNA depletion (Illumina, USA). The starting material (300 ng) of total RNA was rRNA depleted using biotinylated, target-specific oligos combined with Ribo-Zero rRNA removal. The isolated mRNA was subsequently fragmented using enzymatic fragmentation. Then first strand synthesis and second strand synthesis were performed and the double stranded cDNA was purified (AMPure XP, Beckman Coulter, USA). The cDNA was end repaired, 3’ adenylated and Illumina sequencing adaptors ligated onto the fragments ends, and the library was purified (AMPure XP). The mRNA stranded libraries were pre-amplified with PCR and purified (AMPure XP). The libraries size distribution was validated and quality inspected on a Bioanalyzer high sensitivity DNA chip (Agilent Technologies, USA). High quality libraries were quantified using qPCR, the concentration normalized and the samples pooled according to the project specification (number of reads). The library pool(s) were re-quantified with qPCR and optimal concentration of the library pool used to generate the clusters on the surface of a flowcell before sequencing on Illumina HiSeq2500 instrument using hiSeq v.4 reagents (51 cycles) (Illumina, USA.). Experiment setting was 30 million 50 bp paired-end reads. Exiqon data analysis pipeline based on the Tuxedo software package, employing software developed internally at Exiqon to interpret and improve the readability of the final results. The components of our NGS RNA seq analysis pipeline include Bowtie2 (v. 2.2.2), Tophat (v2.0.11) and Cufflinks (v2.2.1). Annotation was done using Mus musculus Reference genome GRCm38, Annotation reference: Ensembl_81. The data is available in NCBI’s Gene Expression Omnibus64 [45] accessible through GEO Series accession number GSE174483 (https://www.ncbi.nlm.nih.gov/geo/query/acc.cgi?acc=GSE174483).

### Global Run On -sequencing (GRO-seq)

Global Run On -sequencing was performed for C166 cells according to previously published protocol [46]. For the analysis of *Vegfa* promoter transcription, publicly available GRO-seq datasets from seven other cell types were used (3T3L1 [47], GEO: GSE56747; AtT20 [48], GEO: GSE64515; B-cell [49], GEO: GSE62296; Liver [50], GEO: GSE59486; MEF [51], GEO: GSE27037; Muscle [52], GEO: GSE26512; Neuron [53], GEO: GSE66703).

### Biotin-labeled miRNA pulldown

C166 cells were transduced with LV-prox and cultured for three weeks before beginning of biotin pulldown experiment. Cells were cultured to 10 cm plates (1,5x 10^6^ cells/plate) on day 1. On day 2, medium was changed and transfections with biotin-labeled miRNAs (mmu-miR-466c-3p ID: HRIZN-009055; mmu-miR-466c-5p ID: HRIZN-009047; cel-miR-67 (negative control siRNA) ID: HRIZN-009049; Dharmacon/Horizon Discovery, UK) were done by using TransIT-TKO® Transfection Reagent (Mirus Bio, USA) according to the manufacturer’s instructions using final concentration of miRNAs 40 nM (280 pmol) and five replicates. On days 3 and 4, pulldown protocol was done according to the protocol of Wani & Cloonan [54]. Apart from the protocol, 50 μl of Dynabeads MyOne Streptavidin C1 magnetic beads (Thermo Fisher Scientific) were used per one sample. RNeasy Mini Kit (Qiagen, Germany) was used to extract RNA. Elution was done by eluting twice with 30 μl of molecular biology water. 8 μl of each sample was used for DNase treatment and cDNA synthesis like previously described.

### Luciferase assay

*Vegfa* promoter sequence (-538 − -344 bp relative to TSS) was ordered as duplex DNA oligo from IDT (S1 Table) and was cloned into miTarget™ miRNA 3’ UTR Target Clone plasmid (MmiT028449-MT06, GeneCopoeia, USA). HEK293T cells were seeded at a density of 100 000 cells/well on 12-well plate. The following day, cells were co-transfected with mimics (mmu-miR-466c-3p ID: MIMAT0004878; mmu-miR-466c-5p: MIMAT0004877; negative control siRNA cat. C-121964-00-20; Dharmacon) and miTarget™ miRNA 3’ UTR Target Clone plasmid containing the *Vegfa* promoter (S1 Table) or negative control vector plasmid (CmiT000001-MT06, GeneCopoeia). Transfection was done using co-transfection protocol from TransIT-TKO transfection reagent according to manufacturer’s instructions (TransIT-TKO (Mirus Bio) for miRNA, TransIT-2020 (Mirus Bio) for plasmid), where 1 μg of plasmid DNA was transfected to the cells by using 3 μl of TransIT-2020 transfection reagent. After 24h, cells were washed once with PBS, collected, and used for luciferase assay. Luciferase assay was performed using Luc-Pair™ Duo-Luciferase Assay Kit 2.0 (GeneCopoeia) according to manufacturer’s instructions. Luminescence measurements were performed using CLARIOstar plate reader (BMG Labtech, Germany) with emission wavelength of 580 nm for Firefly Luciferase and 480 nm Renilla Luciferase. Results are presented as the ratio of RLU(Firefly): RLU(Renilla).

### Statistics

Statistical significance was assessed by one-way ANOVA with Bonferroni post hoc correction or unpaired two-tailed t-test with Welch’s correction as appropriate (GraphPad Prism 5 (GraphPad Software, USA)). Outliers were tested using GraphPad’s Outlier calculator (https://www.graphpad.com/quickcalcs/Grubbs1.cfm). Data are presented as mean±SD. Differences were considered significant when P≤0.05.

## Supporting information

S1 Fig Unmodified blots. Uncropped and unadjusted images of western blots (used to generate data blots in Fig 1C). Blots were imaged with the ChemiDoc MP Imaging System (Bio-Rad).
(TIF)

S2 Fig GRO-seq of promoter ncRNA. GRO-seq data from other mouse cell lines show promoter-associated ncRNA at *Vegfa* promoter in other cell lines as well.
(TIF)

S1 Table Primers and oligos. Primers and oligos used in this paper are listed on the table.
(XLSX)

S1 Raw images (PDF)
