# Supplementary figures and images for: Nuclear microRNA-466c regulates Vegfa expression in response to hypoxia
Source: PLoS One. 2022 Mar 31;17(3):e0265948. doi: 10.1371/journal.pone.0265948 (PMC8975276; doi:10.1371/journal.pone.0265948)

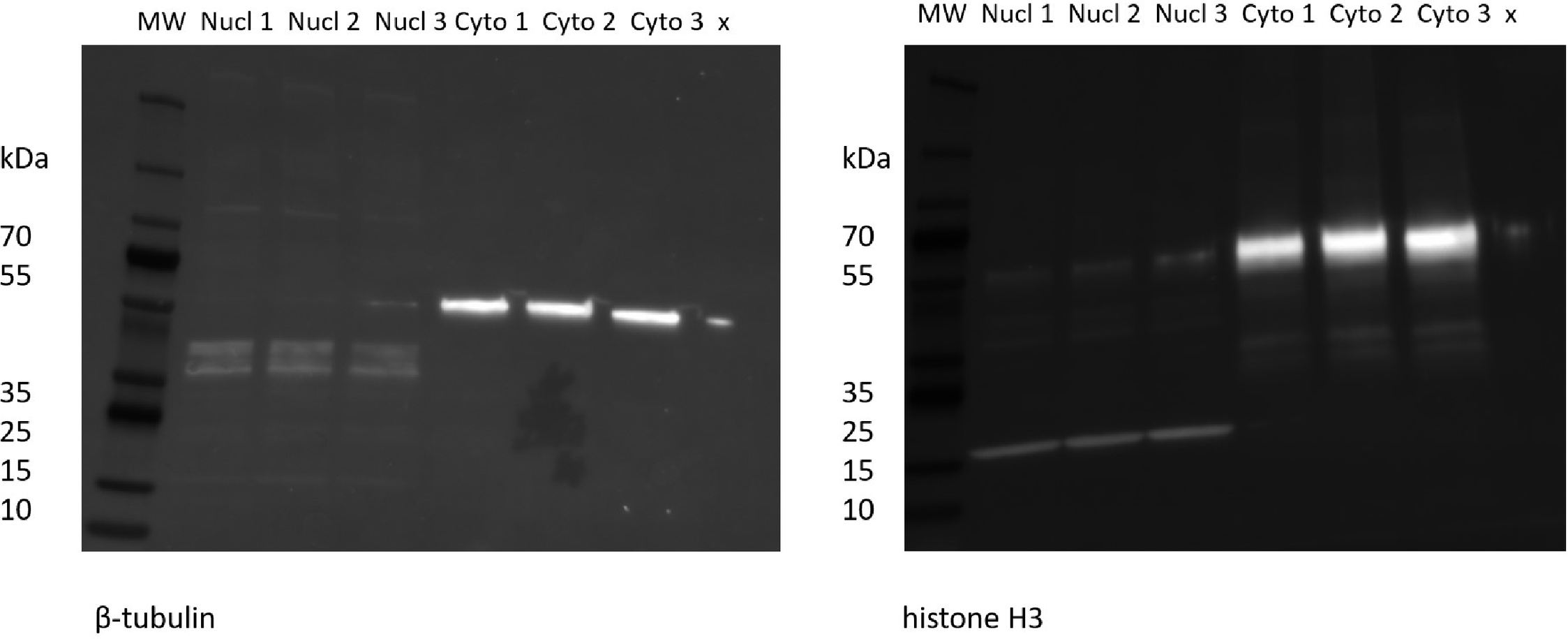

Supplement: S1 Fig — Uncropped and unadjusted images of western blots (used to generate data blots in Fig 1C). Blots were imaged with the ChemiDoc MP Imaging System (Bio-Rad). (TIF) [file pone.0265948.s001.tif]

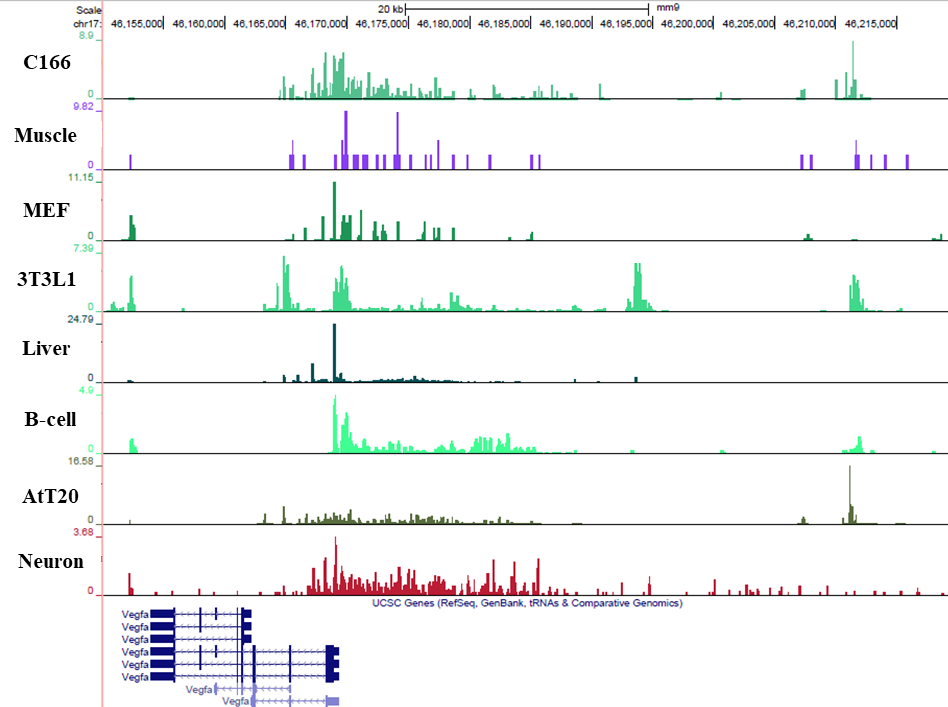

Supplement: S2 Fig — GRO-seq data from other mouse cell lines show promoter-associated ncRNA at Vegfa promoter in other cell lines as well. (TIF) [file pone.0265948.s002.tif]

MW Nucl 1 Nucl 2 Nucl 3 Cyto 1 Cyto 2 Cyto 3 x

kDa

70

55

35

25

15

10

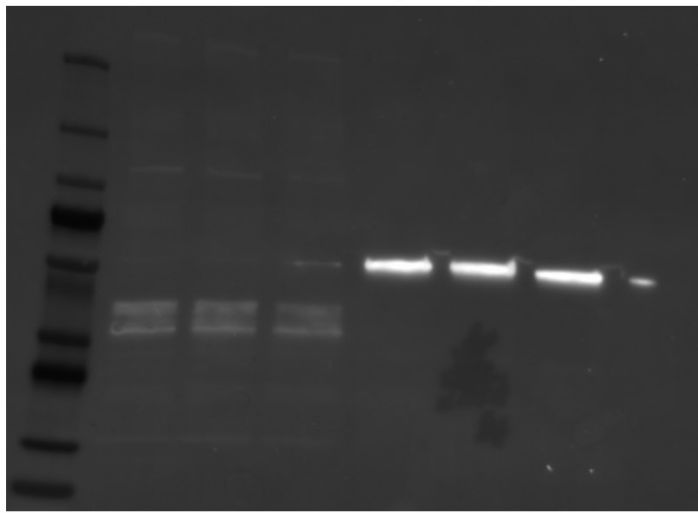

$\beta$ -tubulin

MW Nucl 1 Nucl 2 Nucl 3 Cyto 1 Cyto 2 Cyto 3 x

kDa

70

55

35

25

15

10

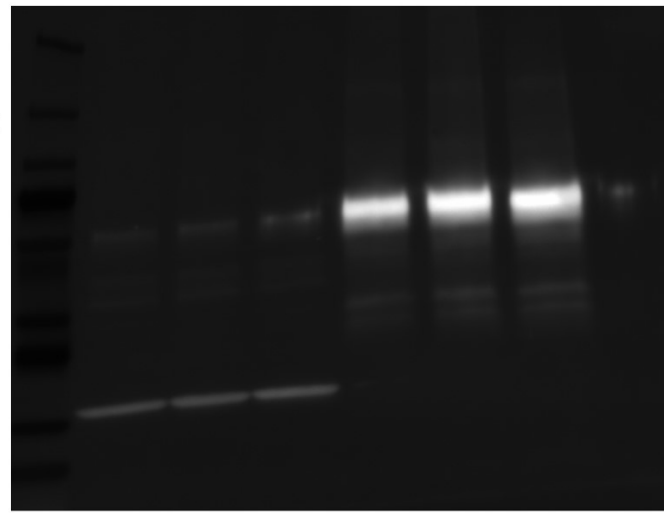

histone H3

Supplement: S1 Raw images — (PDF) [file pone.0265948.s004.pdf]
